# The Health-Related Quality of Life and Putative Factors of Icelandic and American Youth with Multiple Disabilities Including Visual Impairments: A Preliminary Investigation

**DOI:** 10.3390/children13030351

**Published:** 2026-02-28

**Authors:** Ali Brian, Andrea Taliaferro, Pamela Beach, Benjamin Lytle, Adam Pennell, Lauren Lieberman, Ingi Einarsson

**Affiliations:** 1 Department of Educational and Developmental Science, College of Education, University of South Carolina, Columbia, SC 29208, USA; taliafa@mailbox.sc.edu; 2 College of Health Sciences and Technology, Rochester Institute of Technology, Rochester, NY 14623, USA; psbchst@rit.edu; 3 Department of Teaching, Learning, and Teacher Education, University of Nebraska-Lincoln, Lincoln, NE 68588, USA; blytle@lps.org; 4 Natural Science Division, Pepperdine University, Malibu, CA 90263, USA; adam.pennell@pepperdine.edu; 5 Kinesiology, Sport Science, and Physical Education, SUNY-Brockport, Brockport, NY 14420, USA; llieberman@brockport.edu; 6 School of Science and Engineering, University of Reykjavik, 102 Reykjavík, Iceland; ingithore@ru.is

**Keywords:** physical activity, well-being, cross-cultural comparison, assessment, self-perception

## Abstract

Background/Objectives: Health-related quality of life (HRQoL) is a critical indicator of developmental progress, educational engagement, and psychosocial resilience. By identifying both shared and context-specific differences in HRQoL, we aim to contribute to a more nuanced understanding of well-being that can inform the development of assessment approaches and future research tailored to the diverse contexts in which children with disabilities live and learn. Thus, the purpose of this study is to explore HRQoL and its putative influencing factors among youth with multiple disabilities across two distinct cultural settings, the United States and Iceland. Methods: Participants (*N* = 26; Icelandic = 50%; *Mage* = 16.34 ± 2.33 years) completed height, weight, the Test of Perceived Physical Competence (TPPC), Supine-to-Stand (STS), Rapid Assessment of Physical Activity (RAPA), and VISIONS QL. We conducted five, 2 group × 2 sex ANOVA and several independent samples *t*-tests within groups by sex for our variables of interest. Results: There was a significant difference between Icelandic boys and girls for BMI (*p* = 0.087, *d* = 0.65) and STS (*p* = 0.027, *d* = 1.04). Conversely, a significant difference was found in the American group between boys and girls for RAPA (*p* = 0.092, *d* = 0.81) and TPPC (*p* = 0.068, *d* = 0.92). Conclusions: Preliminary findings suggest that patterns in objective and self-reported health indicators may vary by context. These results highlight the importance of considering both measured performance and self-perceived health when examining HRQoL among adolescents with multiple disabilities, while underscoring the need for further research in larger samples to clarify these relationships.

## 1. Introduction

Health-related quality of life (HRQoL) is a multidimensional construct encompassing physical, emotional, social, and functional well-being [1]. In pediatric populations, HRQoL serves as a critical indicator of developmental progress, educational engagement, and psychosocial resilience [2]. For youth with multiple disabilities, including visual impairments, HRQoL is often compromised due to compounded challenges in mobility, communication, peer interaction, and access to inclusive environments [3,4].

Visual impairment affects approximately three percent of children in the United States and is frequently accompanied by additional disabilities such as cognitive delays, motor impairments, or sensory processing disorders [5]. These co-occurring conditions can exacerbate barriers to independence and inclusion, contributing to lower self-esteem, increased anxiety, and reduced opportunities for social participation [6,7]. In Iceland, although the overall prevalence of childhood visual impairment is lower due in part to population size and centralized health registries, youth with multiple disabilities encounter many comparable functional and social challenges [8]. However, the broader contextual environments differ meaningfully between the two countries.

The United States operates within a large, decentralized healthcare and educational system characterized by variability in service access across states, insurance structures, and school districts. Access to specialized services, inclusive recreation, and assistive technologies may depend on geographic location, socioeconomic status, and local policy implementation. In contrast, Iceland has a smaller, nationally coordinated healthcare and education system with universal coverage and centralized disability services. While this structure may facilitate more standardized service provision, the smaller population base can limit the availability of specialized programming and peer networks for children with low-incidence disabilities. These structural differences provide a meaningful context for comparison, as they represent distinct service delivery models, decentralized versus centralized, within high-income countries committed to inclusive education and disability rights [8,9].

Despite differences in healthcare and educational systems, both countries share a growing commitment to improving outcomes for children with disabilities through evidence-based interventions and inclusive policy reform [8,9]. Comparing youth with multiple disabilities across these contexts may therefore help illuminate both shared challenges and context-specific influences on HRQoL. However, cross-national research in this population remains limited, particularly for youth with visual impairments and additional disabilities.

Children with visual impairments often report lower HRQoL compared to their peers without visual impairments, particularly in emotional and social domains [10,11]. However, few studies have examined HRQoL in youth with multiple disabilities across different cultural contexts. Moreover, most QoL instruments used in clinical and educational settings are generic and may not fully capture the nuanced experiences of youth with sensory impairments. Tools such as the Pediatric Quality of Life Inventory (PedsQL) and the Vision-Related Quality of Life Questionnaire (VQoL) have advanced the field, but cross-national psychometric vetting and adaptation remain limited [2,4].

Beyond cultural and systemic factors, individual-level predictors of HRQoL, including putative factors, warrant closer examination. Physical activity has been shown to positively influence emotional well-being, self-esteem, and social connectedness in youth with disabilities [6,12]. Age and sex are also relevant, as developmental stages and gender identity may shape how children perceive their health and social roles [13]. Self-perception of movement, or how children evaluate their own physical competence and mobility, may be especially salient for those with visual and motor impairments [14,15]. This perception can influence both participation and psychological adjustment [7,14,15]. In addition, objective measures of functional mobility, such as the Supine-to-Stand test, offer valuable insight into a child’s physical capability and independence [16]. The Supine-to-Stand test, which assesses the ability to transition from lying to standing, has been linked to motor proficiency, balance, and overall physical functioning in individuals with disabilities, but requires more vetting for children with disabilities [17]. Body mass index (BMI) also plays a role in shaping HRQoL, as an elevated BMI has been associated with reduced physical activity, lower self-esteem, and increased risk of secondary health conditions in youth with disabilities [18]. Furthermore, the level of vision, ranging from mild impairment to total blindness, may directly affect autonomy, participation, and perceived competence [14]. Accordingly, the level of vision is a critical and putative variable in understanding HRQoL outcomes [19].

Given the limited cross-national evidence in this population and the modest sample size (*N* = 26), the present study is framed as exploratory. Rather than testing definitive hypotheses, we aim to generate preliminary insights into HRQoL and selected putative influencing factors among Icelandic and American youth with multiple disabilities, including visual impairments. Specifically, we describe physical activity, age, sex, self-perception of movement, Supine-to-Stand, BMI, level of vision, and HRQoL across the two cultural contexts. We further explore patterns of association and potential cross-cultural differences with appropriate caution. By identifying preliminary shared and context-specific trends, this exploratory work seeks to inform future, larger-scale investigations and the development of culturally responsive intervention and assessment approaches for youth with disabilities that reflect the diverse realities in which children with disabilities live and learn.

## 2. Materials and Methods

### 2.1. Design, Participants, and Setting

This study featured a descriptive–analytic design with convenience sampling. Participants (*N* = 26) included youth and young adults with multiple disabilities (all with visual impairments) from the United States (*n* = 13) and Iceland (*n* = 13) who attended a sports camp for children with visual impairments. The American sample (*Mage* = 16.34, SD = 2.33 years; boys = 5, girls = 8; blind = 2, visually impaired = 11; cognitive impairment = 7) mainly possessed a congenital visual impairment (*n* = 12). The Icelandic sample (*Mage* = 18.08, SD = 4.84 years; boys = 4, girls = 9; blind = 6, visually impaired = 7; cognitive impairment = 9) also mostly included participants with congenital visual impairment etiologies (*n* = 9). Visual impairment is a broad term that includes all levels of reduced vision, even when corrected with glasses or lenses, with a range of mild–severe as well as blindness [20]. For the purposes of this study, blindness is classified as visual acuity worse than 20/200 in the better eye or a visual field of less than 20 degrees, even with correction; visually impaired includes those with visual acuity from 20/40—better than 20/200 in the best eye with correction.

### 2.2. Instrumentation

#### 2.2.1. Test of Perceived Physical Competence (TPPC)

The Test of Perceived Physical Competence (TPPC; [21]) assessed self-perceptions of physical ability in youth with visual impairments aged 9 years and older. The TPPC followed a structured two-decision format adapted from Harter’s Self-Perception Profile for Children [22] or Adolescents [23]. Each item presented two contrasting statements, such as “Some kids are good at running” and “Some kids are not so good at running.” Participants first chose the statement that best describes them, then indicated whether the statement was “really true” or “sort of true.”

The TPPC underwent psychometric vetting to establish its reliability and validity. Brian et al. [21] reported strong internal consistency (McDonald’s Omega = 0.987) and acceptable model fit indices (CFI = 0.95; SRMR = 0.053). Confirmatory factor analysis supported a unidimensional structure. The TPPC is scored from 1 to 4 per item with a total of 6–24 points available (higher scores indicate higher self-perceptions).

#### 2.2.2. Rapid Assessment of Physical Activity (RAPA)

The RAPA, developed by the University of Washington Health Promotion Research Center [24], assessed participants’ physical activity levels. The RAPA is a brief, self-administered questionnaire consisting of nine items divided into two sections: RAPA1 evaluates aerobic activity, while RAPA2 assesses strength and flexibility behaviors. Participants responded to statements that reflect their current activity patterns, such as walking, engaging in moderate or vigorous exercise, and performing strength or flexibility training. The RAPA1 score ranges from 1 to 7, categorizing individuals from sedentary to regularly active. RAPA2 scores range from 0 to 3, indicating engagement in strength and/or flexibility activities. The RAPA demonstrates sound psychometric properties, including strong test–retest reliability, with reported intraclass correlation coefficients (ICC) ranging from 0.94 to 0.996 across validation studies and weighted kappa coefficients generally exceeding 0.67 ([24,25]). Concurrent validity has been supported through moderate correlations with established physical activity measures (*r* ≈ 0.54–0.59), a sensitivity of 81%, and a positive predictive value of 77% [24,25,26].

#### 2.2.3. Supine-to-Stand (STS)

We used the Supine-to-Stand test (STS) to assess functional mobility in youth and young adults with disabilities, including those with visual impairments. The STS measures the time required to transition from a supine position on a mat to a full upright stance without assistance. To support accessibility, we provided tactile orientation cues and verbal instructions to guide movement initiation and direction. Participants completed three trials, and the fastest time was recorded (denoted as max). Psychometric properties of the STS in pediatric populations indicated strong interrater reliability (intraclass correlation coefficient = 0.90–0.97; [16]) and feasibility across a range of motor abilities [27]. The test has also demonstrated sensitivity to variations in movement patterns, making it a flexible and inclusive measure of gross motor competence [16].

#### 2.2.4. VISIONS QL

The VISIONS Quality of Life (VISIONS QL) instrument is a 63 item measure developed to assess the health-related quality of life in youth with visual impairments [28,29]. Adapted from the HEAR-QL [30], which itself evolved from the PedsQL framework [31], VISIONS QL represents the first targeted tool designed specifically for individuals experiencing vision loss. The instrument encompasses six domains: Educational Implications (9 items); Social Integration (14 items); Psycho-social Well-being (10 items); Speech, Language, and Communication (10 items); Family Relationships (12 items); and General Functioning (4 items). The completion time averages around one hour. Each item is rated on a 4 point scale from 1 (completely disagree) to 4 (completely agree), then converted to a percentage score (25% to 100%). Certain items were reverse scored to account for negatively worded statements (e.g., Q60: “Do you feel independent in daily activities?” vs. Q63: “Do you feel that vision loss causes many restrictions in life?”). Subscale scores were averaged to produce an overall quality of life score, with higher percentages indicating more positive perceptions. The instrument’s development included expert reviews to establish content and face validity, as well as good internal consistency (α ≈ 0.86; ω ≈ 0.86) [28].

### 2.3. Procedures

The Institutional Review Board at University of South Carolina approved all procedures (Pro00110871). Parents provided informed written consent for all participants under 18 years of age. Those aged 18 years or older provided their own informed written consent. Participants were a convenience sample of attendees of a sports camp for those with visual impairments (all attendees were invited to participate). Participants completed all demographics (date of birth, biological sex, degree of vision, multi-morbidities, etc.) with parental assistance (approximately October [Iceland] and July [United States] of 2024). Members of the research staff collected height and weight using a digital scale and portable stadiometer. We then calculated BMI based upon kg/m^2^. Next, participants completed the TPPC, RAPA, and VISIONS QL with trained members of the research staff one-to-one in a quiet location. In Iceland, where necessary, a translator verbally supported respondents who needed surveys to be read aloud. All written documents were translated into Icelandic for those in Iceland who preferred it. Finally, participants completed STS on a gymnasium floor where each of the three trials were digitally recorded. All surveys were triple pass entered by members of the research staff. The STS times were calculated from digital recordings with the maximum time used for all analyses (e.g., the fastest time).

### 2.4. Data Analyses

**Figure 1:**
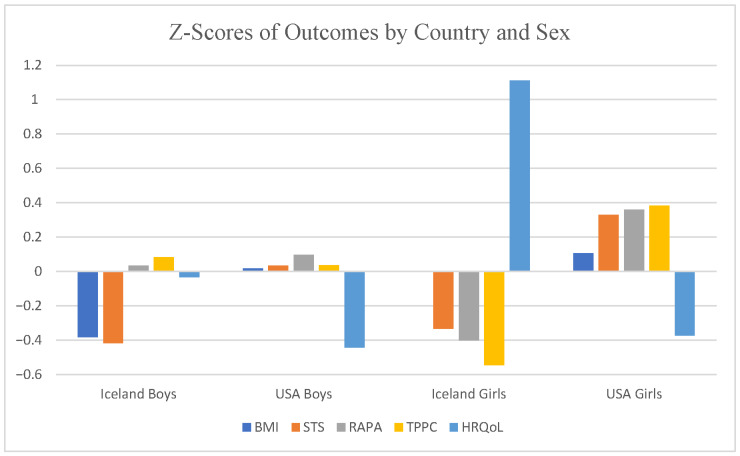
Raw scores were converted into z-scores to standardize the scale for multi-outcome comparison by country and sex. Note: BMI = Body Mass Index; STS = Supine-to-Stand Max Scores; RAPA = Rapid Assessment of Physical Activity; TPPC = Test of Perceived Physical Competence; and HRQoL = Health-Related Quality of Life.

To address our aims, we conducted five, 2 group (USA, Iceland) × 2 sex (boy, girl) ANOVAs for each of our dependent measures. Afterwards, based upon descriptive analyses, we conducted several independent samples *t*-tests within groups by sex. We conducted all analyses via SPSS v 29 (Armonk, NY, USA). Prior to all analyses, we conducted and tested for assumptions via Levene’s test (homogeneity of variance) and Q–Q plots and Shapiro–Wilk (normality). Our first aim, or our descriptives, occurred after all assumption testing and was followed by each ANOVA; alpha was set at *p* < 0.10, given the sample sizes, a priori (see power analyses 2.5). All effect sizes (η_p_^2^ and Cohen’s d) followed Cohen’s interpretations [32] (η_p_^2^ = Small > 0.01, Medium > 0.59, Large > 0.139; Cohen’s d = Small > 0.19, Medium > 0.49, Large > 0.79). For ease of comparison, we transformed all raw data into standardized z-scores as depicted in Figure 1.

### 2.5. Power Analyses

Power analyses were conducted for the planned independent samples *t*-tests and the 2 country × 2 sex ANOVAs. For the *t*-tests, assuming a large effect (Cohen’s *d* = 1.0) and the obtained sample (*N* = 26; *n*_1_ = 13, *n*_2_ = 13), achieved power was approximately 0.72 at α = 0.05 and approximately 0.82 at α = 0.10 (two-tailed). For the 2 × 2 ANOVA, assuming medium effects in the partial eta squared range (ηp^2^ ≈ 0.06–0.09; corresponding to Cohen’s *f* ≈ 0.25–0.31), power with *N* = 25 was below 0.80 at α = 0.05 but approached conventional adequacy under α = 0.10 for medium-to-upper-medium effects. Given the exploratory nature of the study and recruitment constraints, α = 0.10 was selected to balance Type I and Type II error considerations while maintaining transparency through reporting of exact *p*-values and effect sizes.

## 3. Results

All descriptive results are in Table 1. For HRQoL, American boys reported the highest mean (69.62) while American girls the lowest (49.46). Yet, Icelandic boys showed the fastest STS time (1.99 s) and the lowest BMI (22.72 kg/m^2^), which goes against the HRQoL trend. American girls were seemingly the most accurate estimators of their HRQoL with the slowest STS time (4.40 s) and the highest BMI (26.06 kg/m^2^), as well as the most inaccurate with their TPPC and RAPA estimations (being the highest in the sample; see Figure 1).

Regarding our second aim, or the differential impact of country and biological sex on all five outcomes of interest (BMI, STS, RAPA, TPPC, HRQoL), all statistical assumptions were met. The normality of residuals was assessed using the Shapiro–Wilk test and visual inspection of Q–Q plots, confirming that the residuals were approximately normally distributed (*p* > 0.05). The homogeneity of variances was tested using Levene’s test for each dependent variable across all combinations of independent variables. The results indicated that the assumption of equal variances was satisfied (*p* > 0.05). No extreme outliers were detected based on boxplot inspection and standardized residuals. For BMI, the main effects of group (*p* = 0.639, η_p_^2^ = 0.01) and sex (*p* = 0.523, η_p_^2^ = 0.02) were not significant. However, post hoc *t*-tests for the Icelandic group showed a significant difference between boys and girls (*p* = 0.087, *d* = 0.65) that did not occur within the American sample (*p* > 0.10). For STS, the main effects of group (*p* = 0.707, η_p_^2^ = 0.01) and sex (*p* = 0.186, η_p_^2^ = 0.19) were not significant. However, post hoc *t*-tests for the Icelandic group showed a significant difference between boys and girls (*p* = 0.027, *d* = 1.04) that did not occur within the American sample (*p* > 0.10).

For RAPA, the main effects of group (*p* = 0.966, η_p_^2^ = 0.00) and sex (*p* = 0.474, η_p_^2^ = 0.02) were not significant. However, post hoc *t*-tests for the American group showed a significant difference between boys and girls (*p* = 0.092, *d* = 0.81) that did not occur within the Icelandic sample (*p* > 0.10). For TPPC, the main effects of group (*p* = 0.851, η_p_^2^ = 0.002) and sex (*p* = 0.376, η_p_^2^ = 0.04) were not significant. However, post hoc *t*-tests for the American group showed a significant difference between boys and girls (*p* = 0.068, *d* = 0.92) that did not occur within the Icelandic sample (*p* > 0.10). Finally, for HRQoL, the main effect of group (*p* = 0.155, η_p_^2^ = 0.094) was not significant, but the main effect of sex (*p* = 0.025, η_p_^2^ = 0.22) was significant and showed the highest effect size within all tests. Again, post hoc *t*-tests for the American group showed a significant difference (the highest among all analyses) between boys and girls (*p* = 0.003, *d* = 1.90) that did not occur within the Icelandic sample (*p* > 0.10).

## 4. Discussion

The present exploratory study examined the objective and self-reported health indicators among American and Icelandic adolescents with multiple disabilities, including visual impairments. Given the modest sample size (*N* = 26), findings should be interpreted cautiously and viewed as preliminary. Our results revealed distinct patterns in the objective and self-reported health indicators among American and Icelandic adolescents with disabilities. These differences emerged primarily in post hoc comparisons rather than in the main effects of country or sex. The most pronounced disparity occurred in HRQoL, where American boys reported substantially higher scores than American girls (*d* = 1.90). This finding aligns with prior research showing a lower HRQoL among adolescent females with (e.g., [33]) and without disabilities (e.g., [34]); yet, it contrasts with the specific literature on visual impairment that suggests more nuanced sex differences within the limited inquiry (e.g., [3,35].

Like HRQoL, the sex differences in physical activity and motor competence among youth with disabilities were well documented. Boys typically outperformed girls in both objective performance and perceived ability, while girls often reported lower self-efficacy and motivation, which could limit participation and contribute to long-term health disparities [14]. However, the literature focusing on youth with visual impairments revealed a more complex relationship. Although boys still tended to show higher motor competence and activity engagement, perceived competence among girls was more strongly influenced by environmental determinants [36]. These findings could suggest that, for youth with disabilities, contextual and environmental factors may play a more decisive role than biological sex in shaping physical activity patterns and psychosocial outcomes.

This interpretation was supported by our Icelandic sample, where the absence of a comparable sex gap in HRQoL may reflect the influence of national policy and environmental conditions [37]. In Iceland, boys recorded the fastest STS times and lowest BMI, indicating higher functional capacity and lower adiposity, which typically corresponded to favorable cardiometabolic and musculoskeletal profiles [27]. However, these physical advantages did not translate into the highest HRQoL scores, suggesting that physical capacity alone does not fully explain perceived well-being [38].

In contrast, American girls demonstrated the highest RAPA (*d* = 0.81) and TPPC (*d* = 0.92) scores despite having the lowest functional performance and highest BMI. This overestimation of activity and competence aligned with previous findings that adolescents often misjudge their physical activity levels, especially in the absence of objective feedback [39]. Such misalignment may hinder behavior change, as individuals who perceive themselves as sufficiently active may not seek to increase actual activity levels [40].

Sex-specific differences in performance outcomes also varied by country. In Iceland, boys showed slightly lower BMI scores (*d* = 0.65) and significantly faster STS times (*d* = 1.04) than girls, potentially reflecting differences in habitual activity or sport participation [41]. Cross-national data indicated that adolescent boys generally engage in more vigorous physical activity than girls, though the magnitude of these disparities varied by country [40]. In the U.S., sex differences were more evident in self-perception measures and HRQoL than in objective performance, possibly due to the psychosocial factors such as self-efficacy, peer norms, and media influences [42,43,44].

While this exploratory study can provide preliminary cross-national insight into HRQoL and related health indicators among adolescents with multiple disabilities, several limitations must be acknowledged. The small sample size could limit statistical power and generalizability, and the cross-sectional design could preclude causal inference. Reliance on self-reported measures may introduce response bias. Incorporating objective measures of physical activity, such as accelerometry, would reduce reliance on self-reporting and improve measurement accuracy. Qualitative approaches could further explore cultural and environmental factors influencing self-perceptions and performance, providing deeper insight into potential mechanisms underlying observed differences. Longitudinal designs are needed to clarify developmental trajectories and potential directional relationships. Future work should also examine whether physical activity and mobility-related programming that can address both perceived and measured competence could be associated with HRQoL outcomes. Expanding recruitment to additional countries would support broader cross-cultural comparisons and help distinguish universal patterns from context-specific patterns.

## 5. Conclusions

In conclusion, our findings demonstrated that sex-based differences in adolescent health indicators are context dependent and that objective performance and self-perceptions do not always align. Large effect size disparities in HRQoL and self-perception measures in the U.S., contrasted with performance-based differences in Iceland, highlighting the influence of national context on both physical and psychosocial outcomes. Cross-national comparisons must also be approached cautiously. The U.S. and Iceland differ in healthcare structure, educational systems, disability service delivery, and sociocultural norms. While these contextual differences can provide a rationale for comparison, the present sample size does not permit strong inferences regarding systemic influences. Instead, the observed patterns suggest that national context may shape the relationship between functional performance, self-perception, and HRQoL in ways that merit further investigation.

## Figures and Tables

**Table 1:** Demographics for all variables of interest across country and sex.

	HRQoL	STS	BMI	RAPA	TPPC
Iceland					
Boys	54.08 (16.62)	1.99 (0.79)	22.72 (2.29)	8.00 (2.16)	19.75 (3.78)
Girls	50.40 (11.11)	3.62 (1.79)	25.48 (6.18)	8.14 (2.85)	19.57 (3.78)
Total	51.63 (12.54)	3.08 (1.69)	24.46 (5.14)	8.09 (2.51)	19.64 (3.59)
USA					
Boys	69.62 (12.68)	2.26 (0.86)	25.35 (7.23)	7.00 (3.32)	17.40 (5.60)
Girls	49.46 (9.30)	4.40 (5.34)	26.06 (9.72)	8.75 (1.04)	20.87 (2.17)
Total	57.22 (14.30)	3.58 (4.25)	25.78 (8.52)	8.08 (2.25)	19.58 (3.75)
Overall Sample					
Boys	62.71 (15.85)	2.14 (0.79)	24.18 (5.48)	7.44 (2.74)	18.44 (4.75)
Girls	49.94 (9.91)	4.01 (3.87)	25.78 (7.98)	8.47 (2.03)	20.27 (2.99)
Total	54.54 (13.58)	3.34 (3.22)	25.17 (7.06)	8.08 (2.32)	19.58 (3.75)

Note: BMI = Body Mass Index; STS = Supine-to-Stand Scores [fastest time of the three trials]; RAPA = Rapid Assessment of Physical Activity; TPPC = Test of Perceived Physical Competence; and HRQoL = Health-Related Quality of Life. All data are reported in means and (SD).

## Data Availability

The data are not available due to privacy.
